# Thin films nanocomposite: multifunctional materials for energy and water purification

**DOI:** 10.1186/s11671-025-04370-z

**Published:** 2025-11-05

**Authors:** Mahmoud S. Abdel-Wahed, Mohamed H. Sayed, Mohammed M. Gomaa

**Affiliations:** 1 Water Pollution Research Department, National Research Centre, Dokki, Giza, 12622 Egypt; 2 Solid State Physics Department, National Research Centre, Dokki, Giza, 12622 Egypt

**Keywords:** Thin film nanocomposites, Synthesis method, Solar cells, Water treatment

## Abstract

The growing worldwide need for energy and worries about climate change and pollution emphasize the necessity of renewable energy and clean water. Solar energy is among the most abundant and environmentally favorable approaches. Solar cell technology has improved particularly via more effective nanocomposites, making it a viable renewable energy source. Thin film nanocomposite (TFN) offers a promising strategy to address critical renewable energy and water treatment challenges. These innovative materials integrate the unique features of nanoparticles with thin-film architectures to improve performance, durability, and efficiency. TFN generally consists of a matrix material mixed with nanoscale inorganic and/or organic components, providing special characteristics that could enhance the stability and efficiency of solar devices. Obtaining the substantial properties of TFN depends on the compatibility of the mixing components and deposition technique. The utilization of nanomaterials introduces novel functionalities, such as enhanced light absorption, improved charge separation and transport, and increased structural stability, contributing to the overall advancement of solar cell technologies.

Advanced deposition techniques and material engineering enhance the optical, electrical, and catalytic properties, improving energy conversion efficiency in solar cells and effective contaminant removal in water treatment. This review summarises the latest developments in TFNs relating to solar energy and water purification. It illustrates how material design and multifunctional integration can facilitate a unified system for various applications. The review highlights the importance and innovation of TFNs as a sustainable and scalable clean technology solution.

## Introduction

 In contemporary society, two critical challenges are increasingly prominent: the diminishing availability of clean energy resources and the rising demand for safe and accessible water supplies [1–3]. The exhaustion of fossil fuel reserves, coupled with the adverse environmental impacts associated with conventional energy generation, has resulted in energy shortages and has spurred the search for sustainable alternatives. The escalating global demand for sustainable energy solutions has increasingly directed the focus of the scientific community toward the investigation of renewable sources such as geothermal energy, wind power, biomass, and solar energy, as opposed to traditional energy production methods [4]. Concurrently, water scarcity poses significant challenges for billions of people worldwide, with numerous regions struggling to access clean water due to issues such as pollution, climate change, and insufficient treatment technologies. These pressing challenges underscore the urgent need for advancements in technologies that can diminish reliance on conventional energy sources while enhancing water management and purification systems [1, 2].

Thin film nanocomposites (TFNs) have emerged as a promising solution to address both of these issues [5, 6]. These advanced materials, which consist of nanoparticles embedded in a matrix material, offer unique properties that can enhance the efficiency of solar cells and water treatment systems [7]. In solar energy applications, TFNs can increase the absorption and conversion efficiency of sunlight, leading to more effective and cost-efficient solar cells [7]. Meanwhile, in water treatment, nanocomposite films can be tailored for advanced filtration, desalination, and pollutant removal, offering improved performance over traditional materials [8]. As such, TFN presents a transformative technology capable of advancing both energy sustainability and water purification, thus playing a crucial role in solving two of the world’s most pressing problems.

**Fig. 1:**
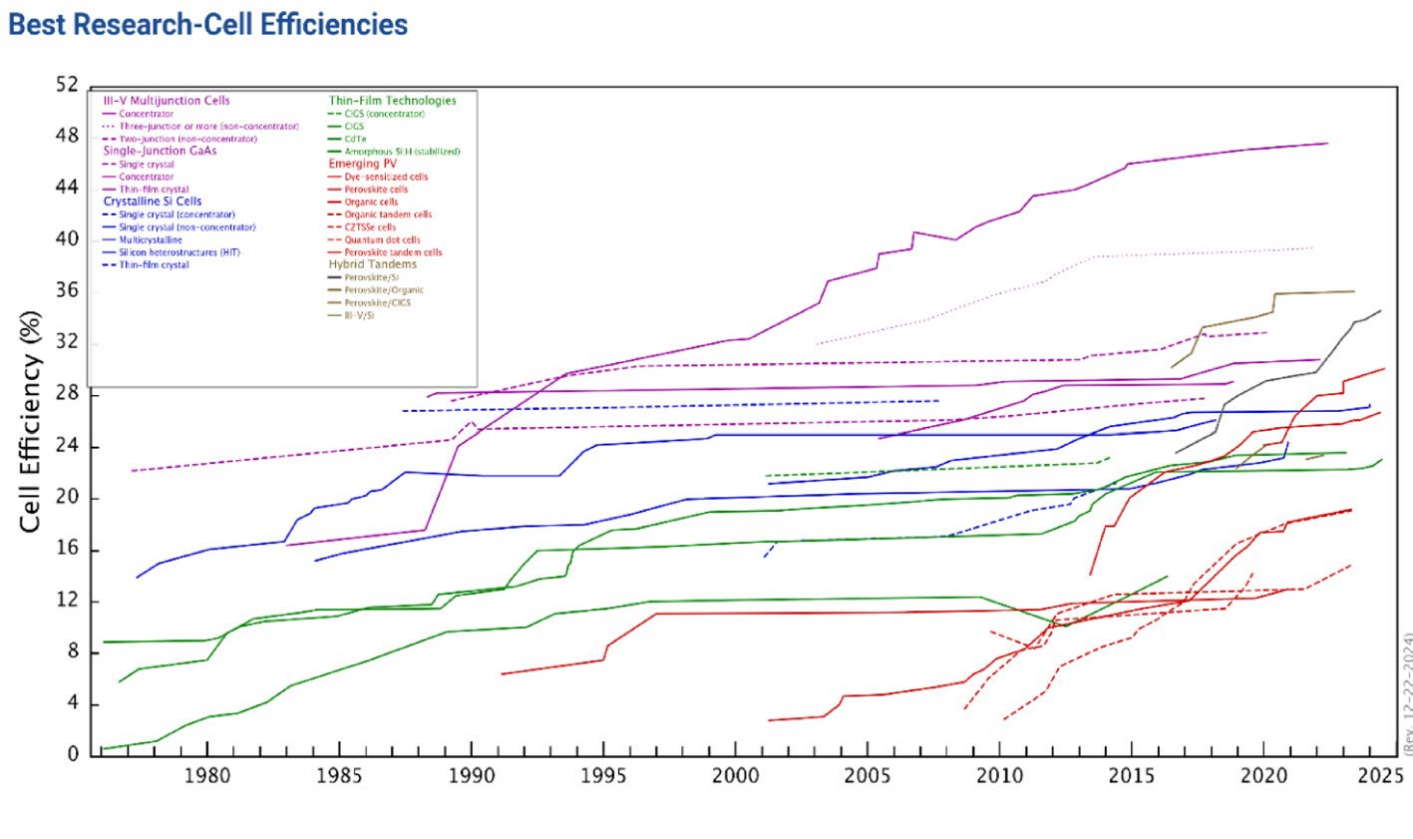
NREL best research-cell efficiencies chart. (Accessed December 2024)

At present, solar energy usage remains limited, accounting for only 0.015% of electricity generation, 0.3% of heat, and 11% of natural biomass photosynthesis. In contrast, fossil fuels fulfill about 80–85% of the world’s energy requirements [9]. Solar power is one of the most available energy sources that can be converted into electricity using solar cells. Modifications in solar cell technologies have been pursued to mitigate issues related to energy consumption and environmental pollution. Solar cells are technological devices that convert sunlight directly into energy. The most significant parameter used to test the characteristics of solar cells is Power Conversion Efficiency (PCE), which is the ratio of incident solar radiation to produced electrical energy, dependent on the properties of the material and manufacturing defects [10]. There have been significant efforts made in the field of solar cells to use efficient and economical materials during the fabrication process, taking into account the necessities for the optimal material, which have a band gap ranging from 1.1 to 1.7 eV with a direct band structure, facile availability, low toxicity, and high solar conversion efficiency [11]. In addition, the enhanced efficacy of solar cells can be accomplished by using efficient strategies to decrease internal losses such as optical, quantum, and electrical inside the solar cell. The National Renewable Energy Laboratory (NREL) provides a database of the greatest confirmed study cell conversion efficiencies for various solar cell technologies as shown in Fig. 1 [12].

The presented efficiency data covers various semiconducting materials employed in solar cell production, including crystalline-Si, GaAs-single junction cells, cells, thin film technologies, multi-junction devices, and emerging solar cell technologies [12]. To achieve different conversion efficiencies in cell components, different methods of fabrication are used in the fabrication of solar cells. The goal of ongoing research is to find novel, easily accessible, non-toxic photovoltaic materials and a repeatable deposition method that can be used for large-scale manufacturing and high photovoltaic conversion efficiency. Organic and inorganic TFNs have been used in solar cells to enhance solar energy harvesting and support in-charge transport processes. These materials provide new approaches to improve optical absorption, flexibility, environmental stability, and efficiency by merging nanoscale components with conventional solar cell matrices. By integrating nanoparticles and nanotubes, nanocomposite solar cells aim to solve problems with conventional solar technology and offer innovative solutions [7].

TFNs also hold great promise for revolutionizing the water treatment process, including purification, desalination, and wastewater management. By incorporating nanoparticles such as metals, carbon based materials, or metal oxides into these films, their surface area and chemical reactivity are greatly enhanced. This enhancement leads to improved filtration efficiency and pollutant removal, as well as effective desalination of seawater. These specially engineered films can be customized to selectively target and eliminate harmful substances like heavy metals, pathogens, and organic contaminants, offering a practical solution for regions struggling to access clean and safe drinking water. Furthermore, the adaptable properties of nanocomposites allow for the development of multifunctional systems that can simultaneously meet both energy and water needs [8].

TFNs represent a class of sophisticated materials that consist of two or more different phases, usually involving a matrix material that incorporates nanoparticles, resulting in films with nanoscale thickness. These films are specifically designed to display improved or novel characteristics by harnessing the synergistic interactions among the nanostructured elements. The unique benefits provided by TFNs arise from their capacity to combine the individual properties of each constituent, resulting in improvements in mechanical strength, electrical conductivity, thermal stability, and optical performance [13].

The structural configuration of nanocomposites allows for fine-tuning of their properties by altering factors such as the size, shape, and distribution of nanoparticles within the matrix. For instance, by incorporating conductive nanoparticles (like metal oxides or carbon-based nanostructures), the thin films can exhibit higher electrical conductivity, which is valuable in electronic and energy applications. Additionally, these films often display high surface area, increased mechanical durability, and resistance to environmental degradation, making them highly suitable for demanding applications such as solar energy conversion and water purification [8].

TFNs are particularly useful in applications where traditional materials fall short, as they offer lightweight, highly functional surfaces with tailored characteristics that are crucial for advanced technology in fields like renewable energy, catalysis, and environmental remediation. These films are a promising area of research due to their versatility, tunability, and potential for multifunctional applications in various high-performance industries.

TFNs are specialized materials that combine a base, or matrix, material with nanoscale fillers, which could include metal nanoparticles, metal oxides, carbon-based nanostructures (like graphene or carbon nanotubes), or polymers. These films typically range in thickness from a few nanometers to several micrometers and are carefully engineered to enhance specific properties by leveraging nanoscale phenomena, such as quantum effects and high surface area-to-volume ratios. Here’s a deeper look at their structural features, properties, and the mechanisms behind their enhancements [14].

As the issues of energy and water scarcity become increasingly severe, TFNs represent a transformative technology that integrates sustainable energy solutions with advanced water purification methods. The potential to optimize these materials for use in solar energy harvesting and water treatment offers significant prospects for improving the quality of life for many, particularly in developing regions where these issues are most acute. This review will explore the fundamental concepts of TFNs, their applications in solar energy systems and water purification, and the latest advancements that are steering us toward a more sustainable and resource-efficient future.

## Solar cell generations

**Fig. 2:**
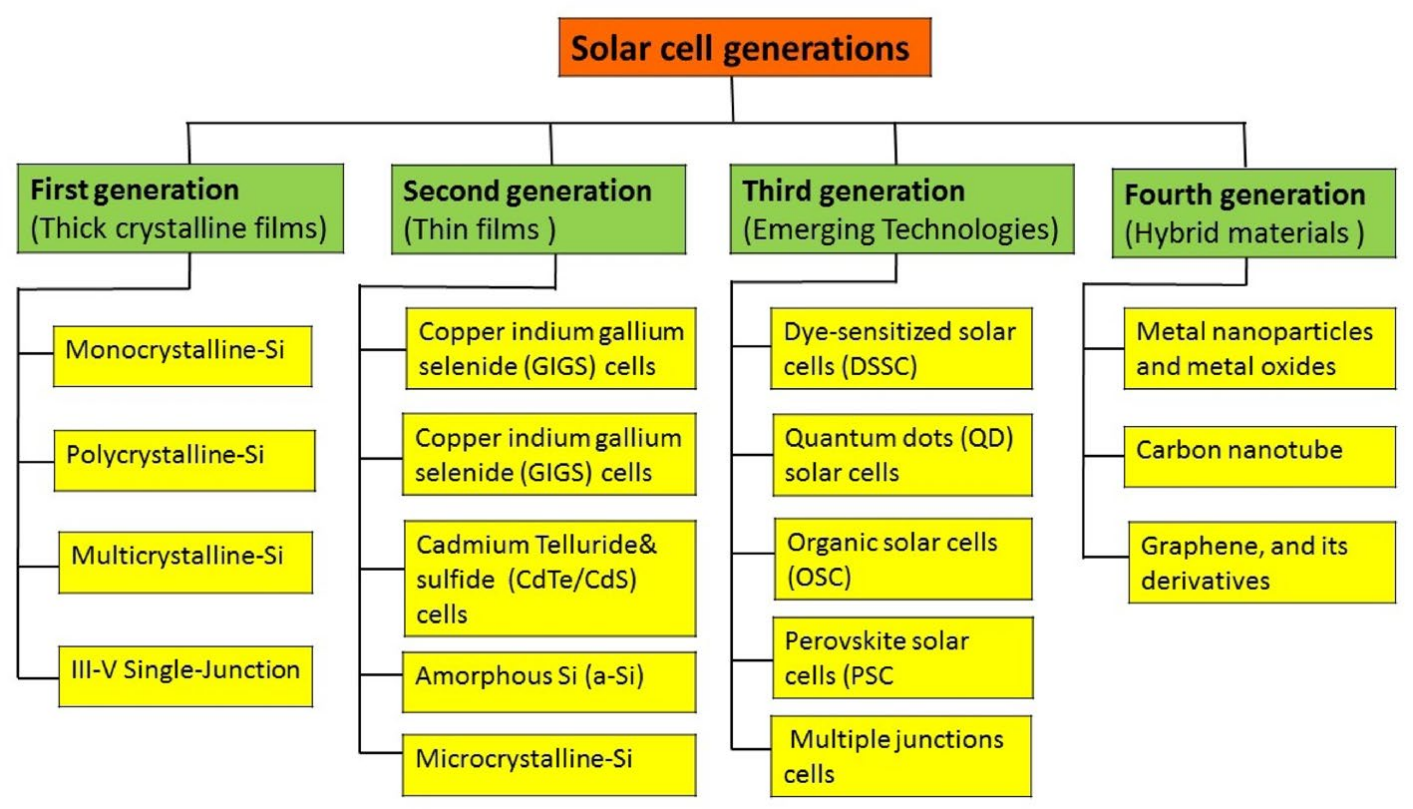
Classification of solar cell technologies and their current technological progress

Solar cell technology is classified into four generations (see Fig. 2), due to the adoption of diverse non-traditional manufacturing methods aiming to innovate functional solar cells [9]. **The first-generation** solar cell technology includes monocrystalline and polycrystalline-Si and monocrystalline-Si, as well as GaAs. Silicon-based solar cells offer stability, high performance, and long service life. However, they come with drawbacks such as expensive manufacturing, sensitivity to temperature, and a rigid, inflexible nature. **The Second Generation** of solar cells involves progress in first-generation technology and the growth of thin film technology, including materials such as µc-Si and a-Si as well as CIGS and CdTe/CdS and inorganic compound-based thin film solar cells. CdTe/CdS solar cells provide a high absorption rate and need less material for fabrication, but they have lower efficiency and pose concerns due to the extreme cadmium’s toxicity. Conversely, CIGS-based solar cells require less material for fabrication but are expensive, lack stability, and are unreliable. **The third generation** includes.

solar cell technology that use nanostructured materials and nanocomposites, as well as nanocrystalline films, Quantum Dots (QDs) [15], Dye-Sensitized Solar Cell (DSSCs), perovskite, multi-junction and organic polymer-based solar cells (OSCs). These solar cells are constructed of semiconducting polymers and colloidal nanostructured semiconductors that have a wide optical absorbance in conjugated polymers and tunable optical band gaps. In this generation, multi-junction solar cells offer the highest efficiency (_~_ 47%) [16], but their fabrication is expensive and complex. Furthermore, OSCs have garnered extensive research focus, both in academia and industry. This is attributed to the simplicity of device fabrication using solution-process able roll-to-roll techniques, which holds the potential to reduce costs and enable large-scale production of OSCs [17]. **The fourth generation** combines the cost-effectiveness and low flexibility of polymer films with the durability provided by innovative nanostructured inorganic materials including metal oxides and metal nanoparticles, as well as nanoparticles with an organic base like graphene (Gr), carbon nanotube (CNT), and Gr-derivatives [11, 18]. Graphene shows potential as an anode in organic solar cells, replacing indium tin oxide (ITO). This is owing to its superior conductivity and transparency. However, it has low hydrophilic characteristics, which adversely impacts the manufacturing process in solution [19]. The incorporation of nanoscale elements into solar cell structures, known as **nanocomposites**, offers unique advantages in enhancing the performance and efficiency of solar energy conversion. This innovative approach harnesses the characteristics of nanoparticles to overcome limitations seen in previous generations, paving the way for the creation of more efficient, cost-effective, and ecologically beneficial solar cells. Therefore, nanocomposites are leading the way in solar cell research and development [20, 21]. Moreover, nanocomposites allow for the engineering of materials at the nanoscale, providing increased surface area and light-trapping capabilities, thus enhancing the absorption of sunlight and offer improvements in electron transport within the solar cell structure. The introduction of nanoscale elements facilitates efficient movement of electrons and holes, reducing recombination losses and enhancing the overall electrical conductivity of the device. These advancements are essential for achieving higher conversion efficiencies in solar cells. However, challenges persist, such as ensuring the stability and durability of nanocomposite materials in diverse environmental conditions. Additionally, issues related to large-scale production and integration into existing infrastructure need to be addressed for widespread adoption. In addition to their functional benefits, nanocomposites provide variety in terms of material selection and production processes. Researchers can modify the characteristics of TFN by selecting appropriate matrix materials and nanofillers, allowing for adaptation for particular solar cell applications [21]. In terms of solar cell-based on nanocomposite categorization, three distinct groups emerge inorganic (metal oxides and nanoparticles), organic (polymers, carbon-based nanotubes, and organic nanoparticles) [22], and hybrid solar, which combines both organic and inorganic components [23]. Each category shows a distinct approach to utilizing nanocomposites to enhance solar energy conversion [19]. Flexible perovskite solar cells (FPSCs) have achieved notable progress, attaining power conversion efficiencies of approximately 26.1% and 29.5% in perovskite Solar Cells (PSCs) and PSCs-tandem cells, respectively, through the utilization of nanocomposite materials [17]. Considering the quantum impact and size-dependent features of nanoparticles in nanocomposite films reveals their influence on solar cell technologies. These aspects include great corrosion resistance, non-toxicity, optical, mechanical, electrical, and magnetic properties [7]. Gr-doped CuO-ZnO TFN with high absorbance coefficients have been used as an important candidate for improving copper indium gallium selenide solar cells (CIGS) [24]. Significant efforts are focused on exploring various nanocomposite materials, with notable improvements in efficiency observed, particularly with nanocomposites such as carbon nanotubes (CNT), TiO_2_, and SiO_2_, yielding the highest PCE [25].

## Structural features of thin film nanocomposites

**Fig. 3:**
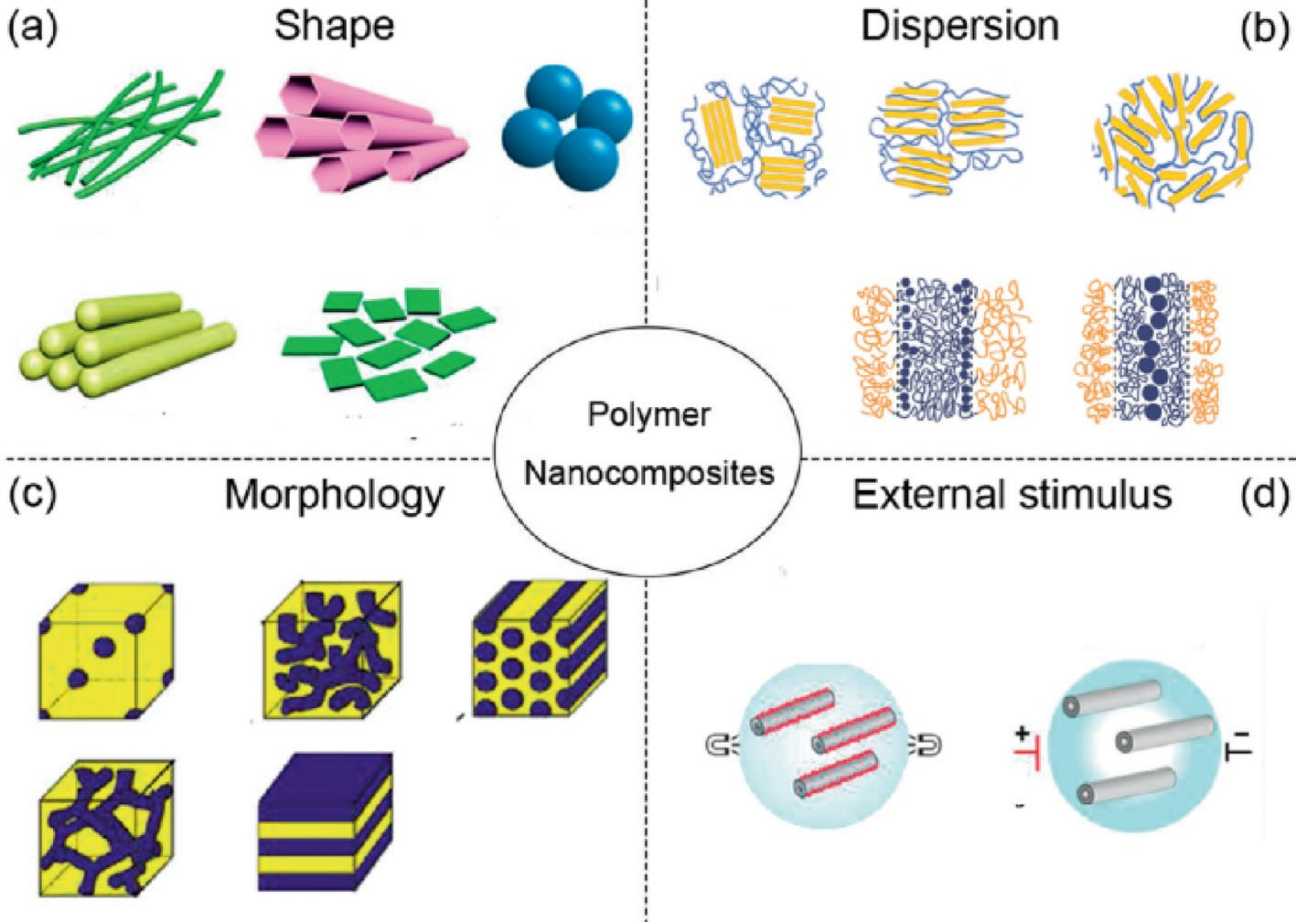
Key factors influencing the structure and properties of polymer nanocomposites [29]

In recent years, scientists have shown increased interest in nanocomposite materials due to their enhanced properties relative to individual metal nanoparticles. Nanocomposites have become the material of the time due to their multi-functionality. Nanocomposites, a growing category of materials, exhibit exciting advancements in areas like enhancing the mechanical and thermal properties of polymers, electrical conductivity, and biological activity. Researchers around the world are working hard to develop new ways to process nanocomposite mixtures to produce nanocomposites with interesting properties [26]. This field aims to improve the practical application of nanocomposite effectiveness, efficiency, and durability by combining suitable components for the synthesis of specific nanocomposites [27]. These attractive characteristics result from the synergistic blend of components with unique properties, incorporating both inorganic and organic systems into a single material. Additionally, the material’s overall properties are influenced by the phase morphology of its components and the characteristics of their interfaces. In principle, the term nanocomposite implies a combination of inorganic and organic elements, like inorganic nanoparticles and organic polymers. However, in practice, its definition extends beyond this, encompassing solid materials with at least two phases, where one phase has dimensions in the nanoscale range [27, 28]. As shown in Fig. 3, the mechanical, optical and functional properties of polymer nanocomposites are significantly influenced by their structural properties, such as the shape of the nanofillers, how they are distributed within the matrix, the resulting morphology and how they react to external influences.

Nanocomposites offer several advantages over traditional composite materials, including a high Surface to volume ratio allowing for smaller filler sizes and spacing, enhanced mechanical properties with increased ductility without sacrificing strength, and improved optical characteristics, where light transmission varies with particle size to improve the performance of solar cells by applying nanocomposite coating, the antireflection properties of cells increase [30].

### Classification of nanocomposites

**Fig. 4:**
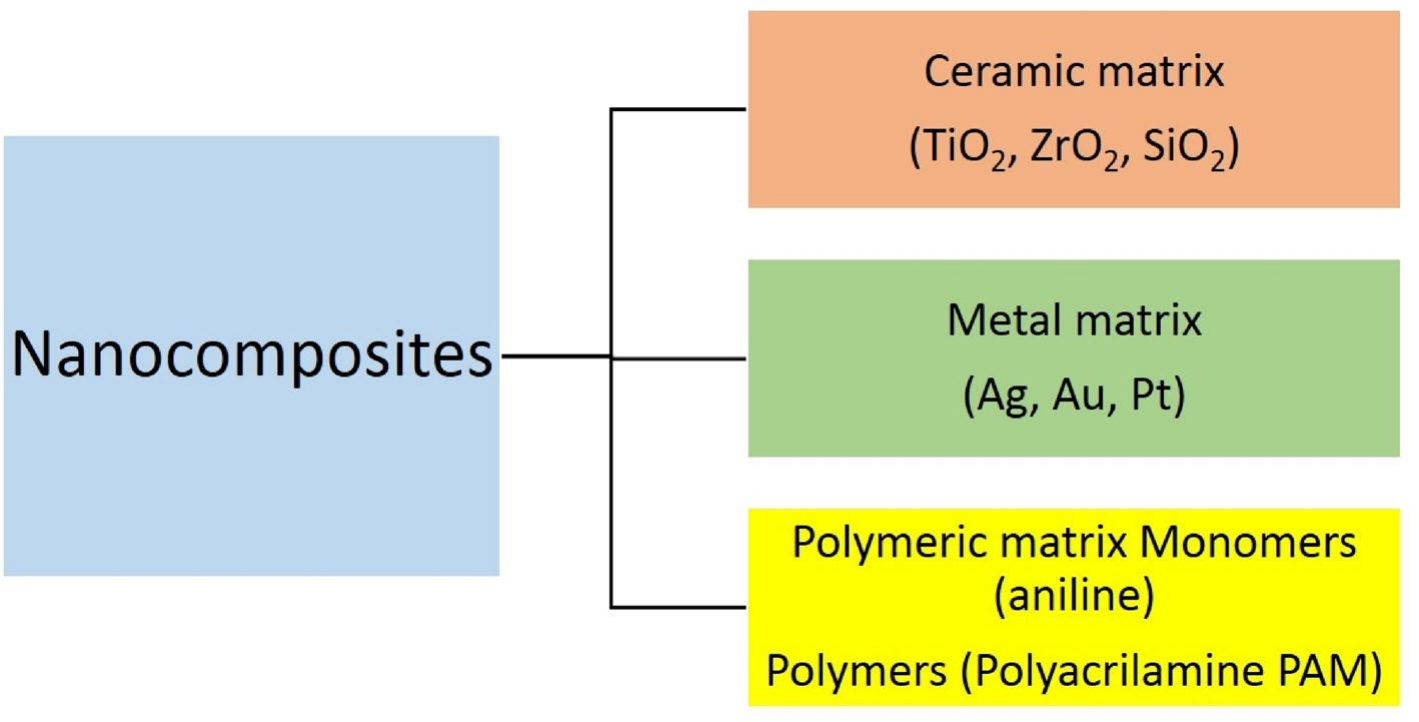
Classification of nanocomposites

Nanocomposite materials are classified based on the presence or absence of polymeric material: non-polymer nanocomposites and polymer nanocomposites. Non-polymer nanocomposites also referred to as inorganic nanocomposites, are those without polymers or polymer-derived materials [20, 24]. They can be further categorized into metal matrix composites, polymer matrix composites, and ceramic matrix composites, depending on the type of matrix material utilized as shown in Fig. 4 [31].

#### Ceramic matrix nanocomposites (CMNC)

Ceramic materials are a diverse group of inorganic, non-metallic solids that are characterised by their remarkable hardness, thermal stability, resistance to corrosion, and low electrical conductivity. These materials are utilized in a variety of structural, electronic, and energy-related applications due to their capacity to endure extreme environmental conditions. Nevertheless, their inherent fragility and limited hardness frequently impede their efficacy. In order to surmount these challenges, ceramic matrices are reinforced with nanoscale materials such as carbon nanotubes (CNTs), graphene derivatives, and metal/metal oxide nanoparticles. This strategy exploits the intrinsic stability of ceramics while concurrently imparting augmented mechanical, electrical, optical, or catalytic properties through nanoscale reinforcement. Consequently, CMNCs have been developed to merge the benefits of ceramics with the distinctive characteristics of nanomaterials, leading to improved performance across various applications. This integration harnesses the advantages of ceramics along with the unique qualities of nanoscale components. The aim is to improve mechanical, thermal, or specific properties for diverse applications. Moreover, various nanocomposites based on transition metal oxides are documented for their valuable and practical applications [32, 33]. A nanocomposite involving CNT-ZnO was utilized as an electrode material for supercapacitors, achieving an enhanced specific capacitance. Additionally, a synthesized graphene oxide GO/ZnO nanocomposite used for photocatalytic degradation of organic contaminants^18^, graphene-ZnO nanocomposite used in photovoltaic cells [34], GO-CuO nanocomposite films as solar absorber layer [35], CNT/CuO nanocomposites films reported for enhancing solar cell absorption [36], and a self-cleaning study involving SiO_2_-modified TiO_2_ composites [21].

#### Metal matrix nanocomposites (MMNC)

Metal matrix nanocomposites (MMNCs) are advanced materials created by integrating nanoparticles into a metal or alloy matrix. The integration process results in materials that demonstrate superior mechanical strength, hardness, thermal stability, and wear resistance when compared to the original metal. The nanoparticles act as reinforcements, impeding dislocation movement and grain growth, thereby enhancing performance in structural applications. The investigation of MMNCs is increasingly common for components that require a high strength-to-weight ratio and durability. The fabrication of MMNCs is generally achieved through the use of vapor-phase deposition and chemical processing techniques. This assertion is corroborated by the extant literature, which includes a documented example of Fe/MgO composites [37].

#### Polymer matrix nanocomposites (PMNC)

Polymer matrix nanocomposites (PMNCs) consist of a polymer matrix that may be either natural (e.g. cellulose or chitosan) or synthetic (e.g. poly(alkylthiophene), polyimides, or polyethylene), which is reinforced with nanoscale additives. At the molecular level, the interaction between the polymer and the nanofillers creates interfacial forces that significantly enhance the material’s properties. It has been demonstrated that even a minimal quantity of nanofillers, when dispersed effectively, can enhance mechanical strength, thermal stability, electrical conductivity, and optical characteristics. Conversely, the phenomenon of agglomeration has been observed to diminish these benefits. In comparison to MMNCs and CMNCs, polymers offer several advantages, including reduced manufacturing costs, decreased density, elevated specific strength, resistance to corrosion, and ease of processing. This results in a reduction in energy consumption during the fabrication and recycling processes. Common inorganic nanofillers include graphene, metal oxides, and carbon nanotubes (CNTs), which enhance performance across a variety of applications, ranging from flexible electronics to membranes for water purification. For instance, poly(alkylthiophene), which possesses a bandgap of approximately 1.9 eV and demonstrates high hole mobility, is frequently employed in organic solar cells. PMNCs can be produced using various methods, including in situ polymerization and sol–gel techniques. These methodologies facilitate the controlled dispersion of nanofillers and the optimisation of multifunctional properties [19, 22, 28, 38].

### Unique properties of thin film nanocomposites

- **Mechanical strength and durability**: TFNs exhibit superior mechanical strength and toughness relative to conventional thin films. The incorporation of nanoparticles into the matrix significantly enhances properties such as hardness, wear resistance, and resilience when subjected to mechanical stress. These enhanced characteristics are particularly vital for applications that demand robust coatings, including solar cells that must withstand outdoor conditions [7].
- **Enhanced electrical and thermal conductivity**: Specific nanoparticles, including silver, carbon nanotubes, and graphene, are known to enhance both electrical and thermal conductivity. This property makes these materials especially ideal for use in electronic and energy-related devices. For instance, conductive nanocomposites are employed in thin-film solar cells to create efficient pathways for electron transport, thereby enhancing charge separation and collection efficiency [7].
- **Optical and photocatalytic properties**: TFNs frequently exhibit adjustable optical characteristics, including a variable refractive index, absorption of UV-Vis light, and photoluminescence. This ability to modify optical properties is crucial in the context of solar cells, as improved light absorption significantly enhances energy conversion efficiency. The enhanced light absorption observed in nanocomposite thin films can be attributed to multiple mechanisms. These mechanisms encompass the following: the localized surface plasmon resonance (LSPR) of embedded metallic nanoparticles, which enhances the local electromagnetic field and increases absorption; light scattering at nanostructured interfaces, which extends the effective optical path length; and bandgap engineering facilitated by the addition of dopants, which permits better alignment with the solar spectrum. The subsequent essay will present a thorough review of the pertinent literature on this topic. Additionally, metal oxide nanocomposites, particularly those based on TiO₂, demonstrate photocatalytic capabilities, rendering them effective in the degradation of organic contaminants for water purification purposes [39].
- **High surface area and porosity**: The nanoscale architecture and high surface area-to-volume ratio of nanocomposite films enhance their surface reactivity, which is a significant benefit in the fields of catalysis and water purification. Additionally, the porous nature of these films promotes efficient mass transport, thereby improving their efficacy in applications that necessitate adsorption or catalytic processes [40].

###  Mechanisms behind property enhancements

- **Quantum effects**: At the nanoscale, materials demonstrate quantum confinement effects that significantly alter their electronic and optical characteristics. For instance, nanoparticles embedded in a matrix can induce a localized surface plasmon resonance (LSPR) effect, especially in metallic nanocomposites. This phenomenon enables the material to selectively absorb certain wavelengths of light, which can be optimized for various applications, including photothermal therapy, solar energy harvesting, and improved photocatalysis for water purification [7].
- **Synergistic interactions**: The incorporation of multiple materials within nanocomposites often produces synergistic effects, where the performance of the composite surpasses the sum of its individual components. The observed effects are attributed to the cooperative interactions among nanoscale constituents. For instance, the combination of a wide-bandgap semiconductor with a narrow-bandgap material has been demonstrated to extend light absorption into the visible or near-infrared region while concomitantly enhancing interfacial charge transfer. The synergistic interactions that ensue are known to result in substantial improvements in overall device performance when compared to the performance of the constituent materials alone. In a similar manner, the incorporation of carbon nanotubes or graphene sheets within a polymer matrix has been demonstrated to enhance electrical conductivity and mechanical strength concurrently. This offers considerable advantages for utilisation in flexible solar cells and electronic devices. The following essay will provide a comprehensive overview of the relevant literature on the subject [7, 41].
- **Reduced recombination rates**: In the context of solar cell technology and photocatalysts process, the utilization of TFNs plays a crucial role in mitigating the rates of electron-hole recombination, a prevalent challenge in photovoltaic systems that adversely affects their efficiency. The integration of nanoparticles that promote charge separation within these films enhances the transport of electrons toward the electrodes, thereby improving the overall power conversion efficiency [7, 41].

## Synthesis techniques of thin film nanocomposites

TFNs can be prepared using combinations of three fundamental building blocks: metals, ceramics, and polymers. The synthesis procedure determines the morphologies, physical and chemical characteristics of the resulting nanomaterials, influencing their potential applications. Researchers are actively exploring cost-efficient, rapid, and environmentally friendly synthesis methods that yield nanomaterials with practical applications and high efficiency [5].

The synthesis methods for nanocomposites are commonly categorized into two classes: the top-down approach, which involves physical methods, and the bottom-up approach, including chemical methods. The bottom-up method involves building up materials from atoms to clusters to nanoparticles. The bottom-up approach is particularly advantageous in nanotechnology and materials science, offering a way to engineer structures at the atomic and molecular levels for enhanced functionality and performance. Common bottom-up methods for nanoparticle synthesis and nanocomposite films include sol-gel, spin coating, dip coating, CVD, spray pyrolysis, hydrothermal, and chemical bath deposition. In the top-down approach, nanoparticles are synthesized by reducing the size, breaking down bulk materials into fine particles [33]. Furthermore, pulsed laser deposition, RF-sputtering, and thermal deposition are among the widely used approaches for nanocomposites and nanoparticle synthesis. The next section has briefly covered common methodologies utilized for the preparation of TFNs that are compatible with solar cell technology [5].

### Spin-coating

**Fig. 5:**
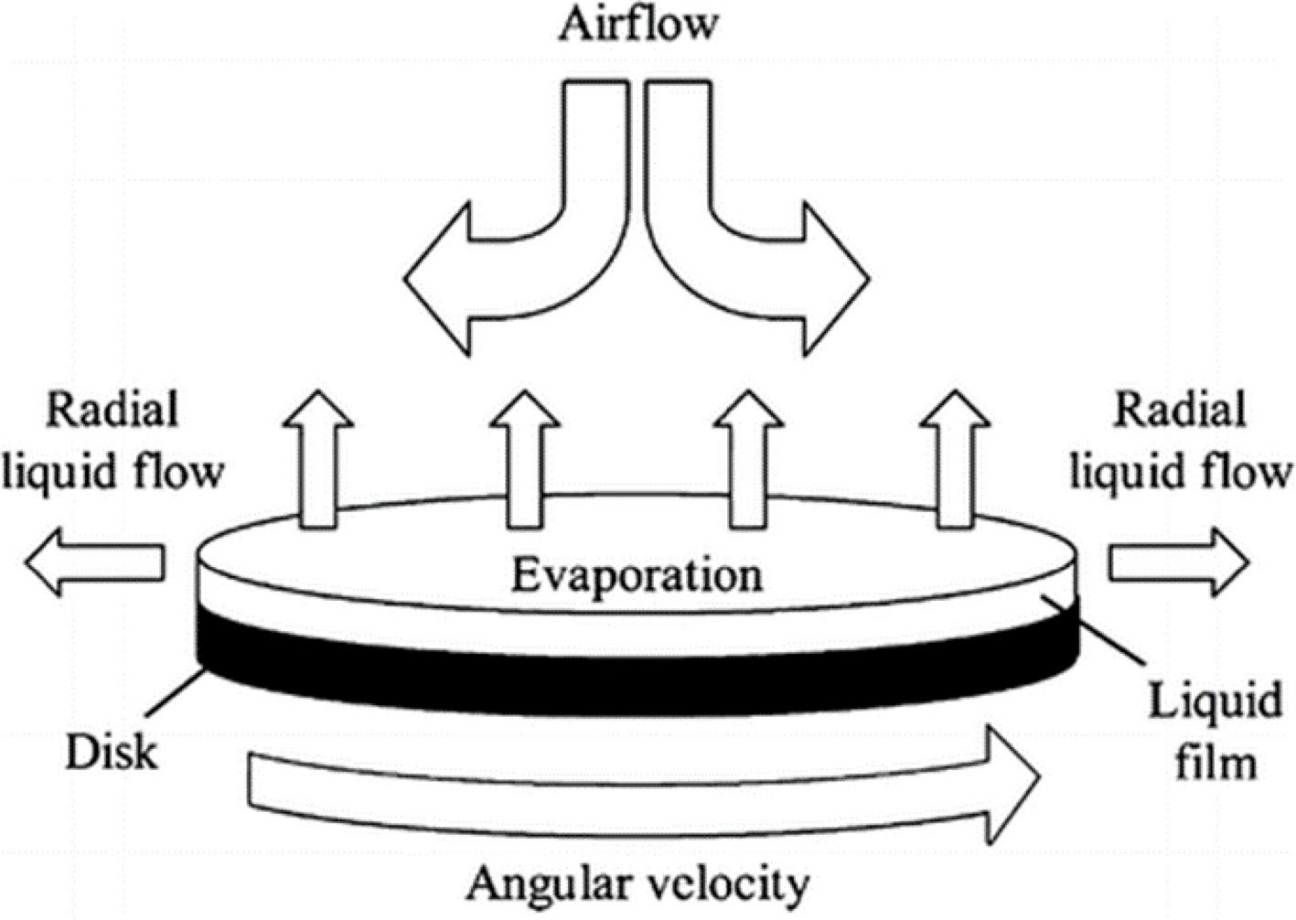
Schematic representation of Spin-coating route [44]

Spin-coating route is widely used as a quick and simple way to deposit films, as shown in Fig. 5. It facilitates the incorporation of various hybrid nanocomposite layers, utilizing nanostructured inorganic materials like metal oxides and chalcogenides, either as absorber layers or buffer layers in solar cell technology. In this process, the organic compound is initially dissolved in a suitable solvent, and the resulting solution is deposited onto a substrate using a dispenser unit. On the substrate, this deposition might take place in a dynamic or static regime. The quantity of solution applied to the substrate is influenced by both its viscosity and the dimensions of the substrate. Subsequently, the substrate undergoes continuous high-speed rotation, facilitating the spreading of the solution through rotational movement and the evaporation of the solvent to achieve the formation of a thin film [19]. Gr-CdS nanocomposite films were created through a combination of chemical bath deposition and spin coating processes. These films were then studied for their properties in the context of CIGS solar cells. To prepare the inks for Gr-CdS nanocomposite films, 0.05 g of the Gr-CdS nanocomposite was dissolved in 10 mL of absolute alcohol. The prepared solution was sonicated for 15 min to achieve a homogeneous preparation [42].

Furthermore. nanocomposite films of TiO_2_/Co-CdS/ZnS were fabricated using spin-coating method. The precursor solutions, consist of TTIP, HCl, and ethanol in a carefully optimized ratio, with stirring for 2h at 50°C. Subsequently, the deposited layer was employed as a photoanode for solar cells [43].

### Chemical bath deposition

**Fig. 6:**
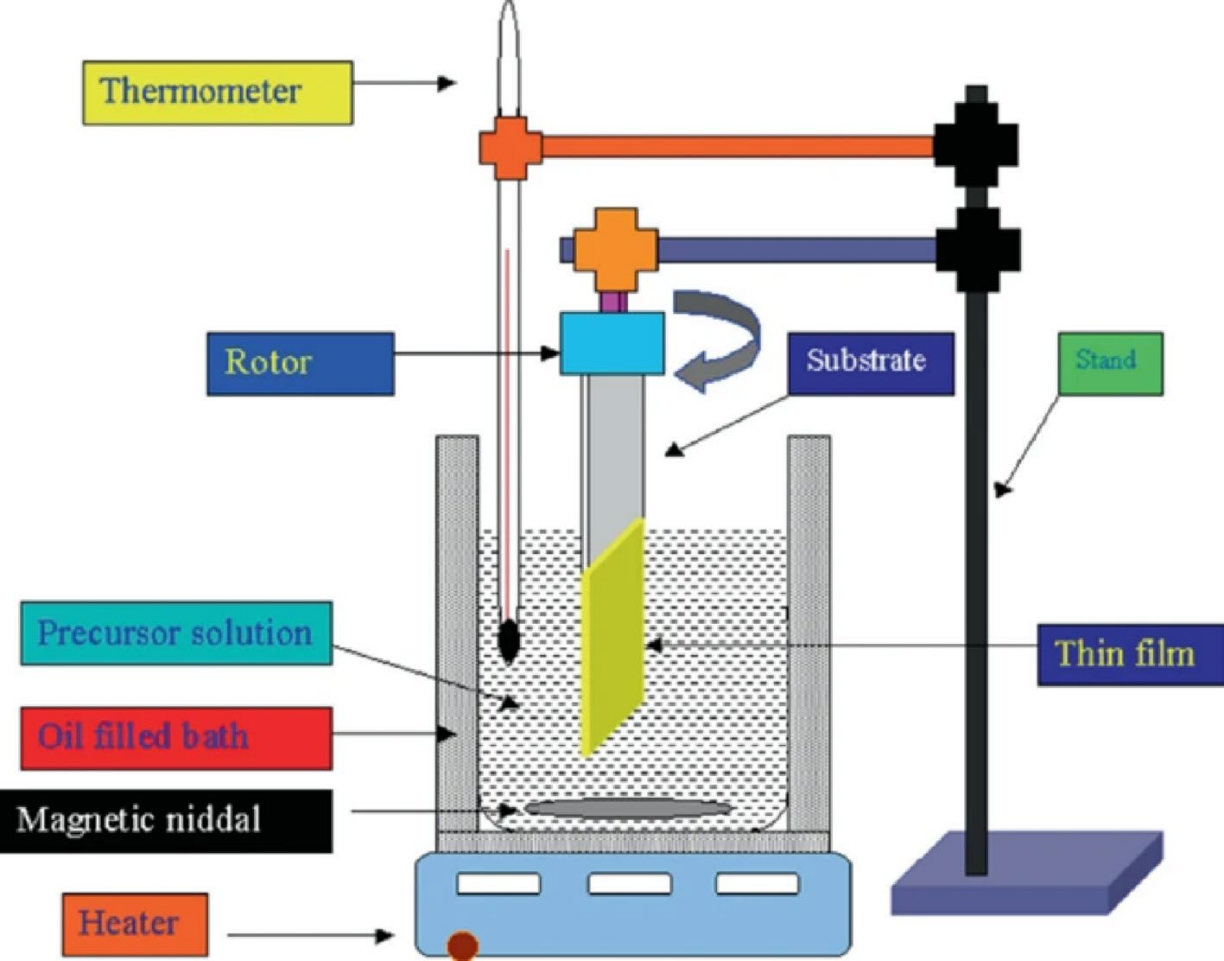
Schematic set up of chemical bath deposition method [49]

The chemical deposition procedure is the simplest strategy for producing various nanomaterials. This approach is based on the interaction of a precursor solution (including chlorides, acetates, sulfites, nitrates, and oxy-chlorides) with complexing agents (such as ammonia, oxalates, EDTA, alkali metal hydroxides, thiourea, and urea) as shown in Fig. 6. To attain the best results, deposition parameters such as pH, deposition temperature, duration time, and starting solution concentration must be optimized for the specific material through the deposition process [45]. While chemical deposition is commonly considered a simple process, it is a complicated physicochemical method with many stages, each having a notable impact on the characteristics of the deposited materials [46]. CdS nanoparticles and Gr-CdS nanocomposite were synthesized using a low-cost chemical bath deposition approach. The process involved using a starting solution of 0.1 M CdSO_4_ and a complexing agent (0.02 M SC(NH_2_)_2_), with 100 mL of a Gr solution. The reaction was maintained at a constant pH of 10. CdS nanoparticles and Gr-CdS nanocomposites were effectively produced at room temperature after a two-hour reaction time. Thin films of Ti/ZnO nanocomposites were deposited using a chemical deposition strategy that involved mixing different amounts of TiO_2_ nanofluid with a zinc solution of solution Zn(NO_3_)_2_ 6H_2_O [47]. *Y. Rodríguez-Lazcano et al.* [48] employed chemical bath deposition and laser ablation to deposit Sb_2_S_3_-Cu TFNs. Sb_2_S_3_ and Sb_2_S_3_-Cu nanocomposites were obtained using a chemical deposition process using a solution of 650 mg SbCl_3_, acetone, and 1 M Na_2_O_3_S_2_, with a total volume of 100 mL. The addition of Cu nanoparticle suspensions produced via laser ablation into chemical solutions at different amounts was explored. This resulted in an improvement in the electrical properties of Sb_2_S_3_-Cu nanocomposites thin films [48].

**Hydrothermal method**

**Fig. 7:**
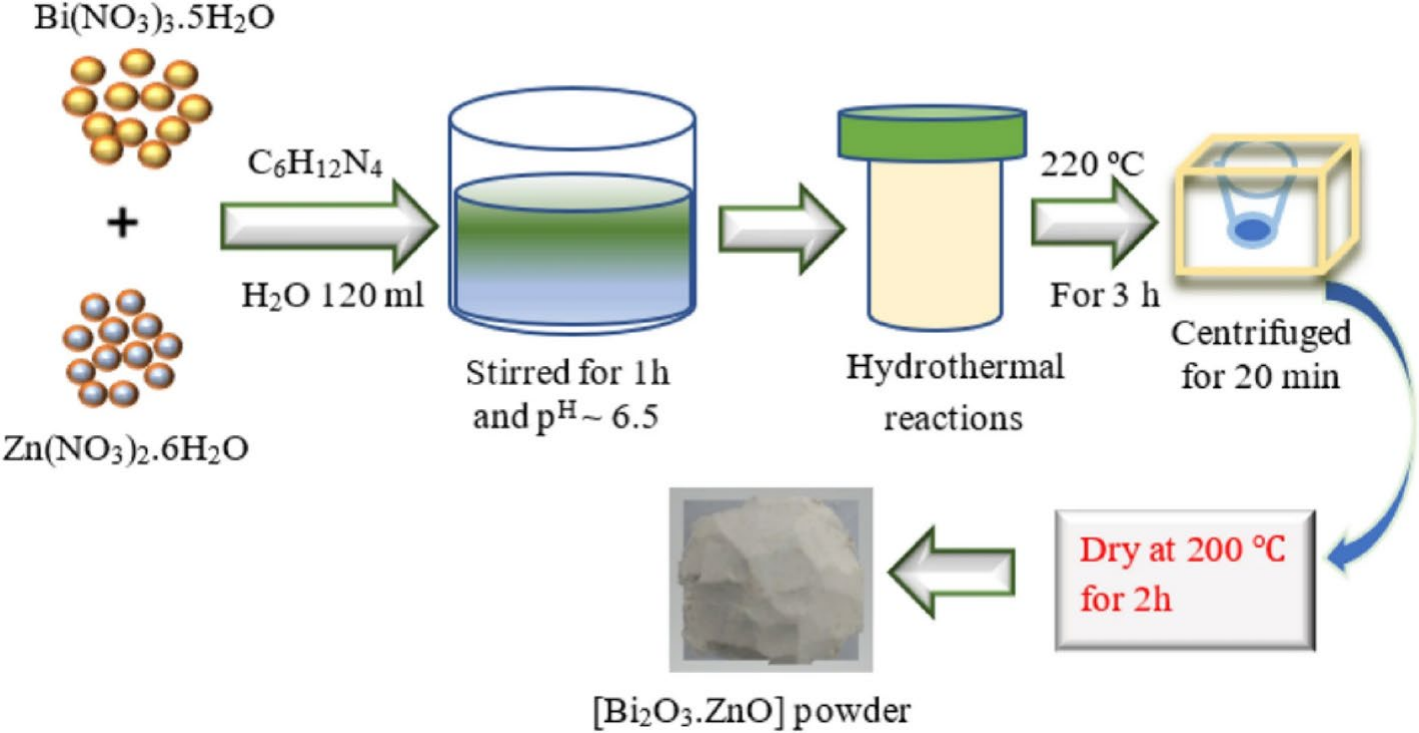
Schematic diagram representation of the hydrothermally prepared nanocomposite material [53]

The hydrothermal method stands out as an easy and affordable alternative compared to other advanced synthesis techniques. This method allows for the direct preparation of nanocomposites thin films using the starting precursor’s solution, with precise control over composition and morphologies as presented in Fig. 7. Hydrothermal approach can employ both inorganic and organic precursors, and in many cases, it eliminates the need for an annealing process. The synthesis takes place at in Teflon-lined, sealed autoclaves at temperatures and pressures above ambient levels, with varying processing times. The hydrothermal process is ecologically beneficial since it involves a closed system and uses solutions as the reaction media [46]. This versatile approach has demonstrated effective in synthesis nanocomposites independently, and its synergy with other techniques has opened new avenues for enhanced materials. Researchers have explored merging of the hydrothermal method with various approaches to tailor the properties of nanocomposites in powder and films form. Ag-C_3_N_4_/ZnWO_4_ nanocomposite with copper-loaded was synthesized through the hydrothermal method, followed by spin coating for thin film fabrication. Initially, ZnWO_4_ nanorods were hydrothermally prepared with Cu nanoparticles, utilizing NaBH_4_ as a reducing agent in the presence of an appropriate amount of g-C_3_N_4_ in an aqueous acid medium. Subsequently, 1 ml of 0.01 M Cu(NO_3_)_2_.6H_2_O solution was introduced to the suspended g-C_3_N_4_ solution [50].

R-GO-TiO_2_ nanocomposites were synthetized via hydrothermal process for fabrication efficient organic solar cells. Ascorbic acid was used as a reducing agent, converting GO to RGO and forming nanocomposites by in situ TiO_2_ deposition on RGO. GO and TiO_2_ nanocomposite were distributed in deionized water and ultra-sonicated. The resultant suspension was then transferred to autoclave with Teflon lining was baked and heated for four hours at 160 °C. The resulting nanocomposite was washed many times before being dried for 12 h in a vacuum oven at 90° C then use for solar cell fabrication [51]. Cds/Te nanocomposites were synthesized hydrothermally using sodium citrate dehydrate, cadmium chloride, and sodium tellurite eques solution. The mixture was then sonicated for fifteen minutes and placed into a Teflon container that was sealed. The reaction container was heated to 180 °C for 40 min, followed by cooling the solution to room temperature. Introducing Zn^2+^ and In^3+^ doping involved adding varying weight percentages of zinc and indium salts, respectively, to the initial solution. Doctor-blade method was employed to transform both pure and doped Cds/Te nanocomposites into photoanode films for dye-sensitized solar cells [52].

### Spray pyrolysis

Spray pyrolysis is a deposition process used to prepare thin films and coatings, including nanocomposite materials. The solution consists of metal salts, organic compounds, or other components that will form the nanocomposite, then the solution is atomized into fine droplets using ultrasonic nebulization or pneumatic atomizer. The small droplets are sprayed over a heated substrate. The fine droplets are sprayed onto a heated substrate. The sprayed droplets decompose and react to form the desired nanocomposite films. Spray pyrolysis is beneficial for nanocomposite films because it provides for homogeneous and controlled deposition on large areas for different applications [54, 55]. *Moez Hajji et al.* [24] utilized the spray pyrolysis technique to fabricate nanocomposite films, both undoped and graphene oxide (GO)-doped CuO-ZnO, on glass substrates. The starting solution involved dissolving zinc acetate in a mixture of distilled water and isopropanol, while the copper solution was created by dissolving copper chloride in distilled water. These solutions were combined in a 1:3 molar ratio of zinc to copper. The deposition process was carried out at a substrate temperature of 350 °C. For the preparation of GO-doped CuO-ZnO films, the same synthesis method was applied, incorporating GO into the solution with varying molar ratios. The resulting GO-doped CuO-ZnO films, produced through spray pyrolysis, were employed to enhance the efficiency of CIGS solar cells [24]. *Rohit Pati et al.* employed an ultrasonic spray method to uniformly deposit CdS-GO nanocomposite films onto conductive substrates for solar cell applications [56]. *V. Janakiraman et al.* [57] developed TFN of Ta_2_O_5_/SnO_2_ using SnCl_2_.2H_2_O at a deposition temperature of 400 °C. The TFN incorporating varying ratios of Ta_2_O_5_ powders, were dispersed into the solution. Notably, the optical band gap had a change from 2.25 eV to 1.95 eV as the Ta_2_O_5_ content increased, exhibiting potential applications in optoelectronic devices [57].

### Chemical vapor deposition (CVD)

**Fig. 8:**
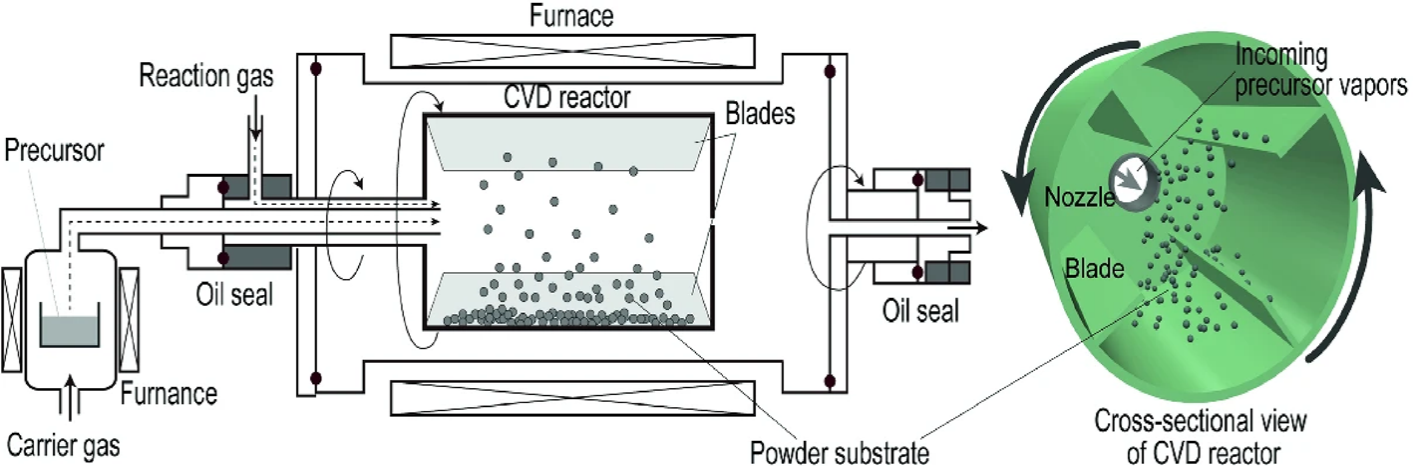
Schematic representation of CVD technique [60]

CVD technique involves depositing vapor generated by heating the precursor material onto a substrate maintained at a relatively lower temperature, as illustrated in Fig. 8. The deposition process is optimized for the use of gas, liquid, or solid precursors to deposit nanocomposites and 2D materials. CVD proves to be an effective approach for producing high-quality nanostructured thin films over large areas [17]. The CVD method has been employed for the growth of MoS_2_/MoO_2_ nanocomposite films through adjustments in the deposition conditions [58]. *Erdi Akman et al.* [59] utilized the CVD technique to enhance the stability and efficiency of Cu_2_S TFN by incorporating graphene for Quantum Dot Sensitized Solar Cells (QDSSC) [59].

### RF sputtering

**Fig. 9:**
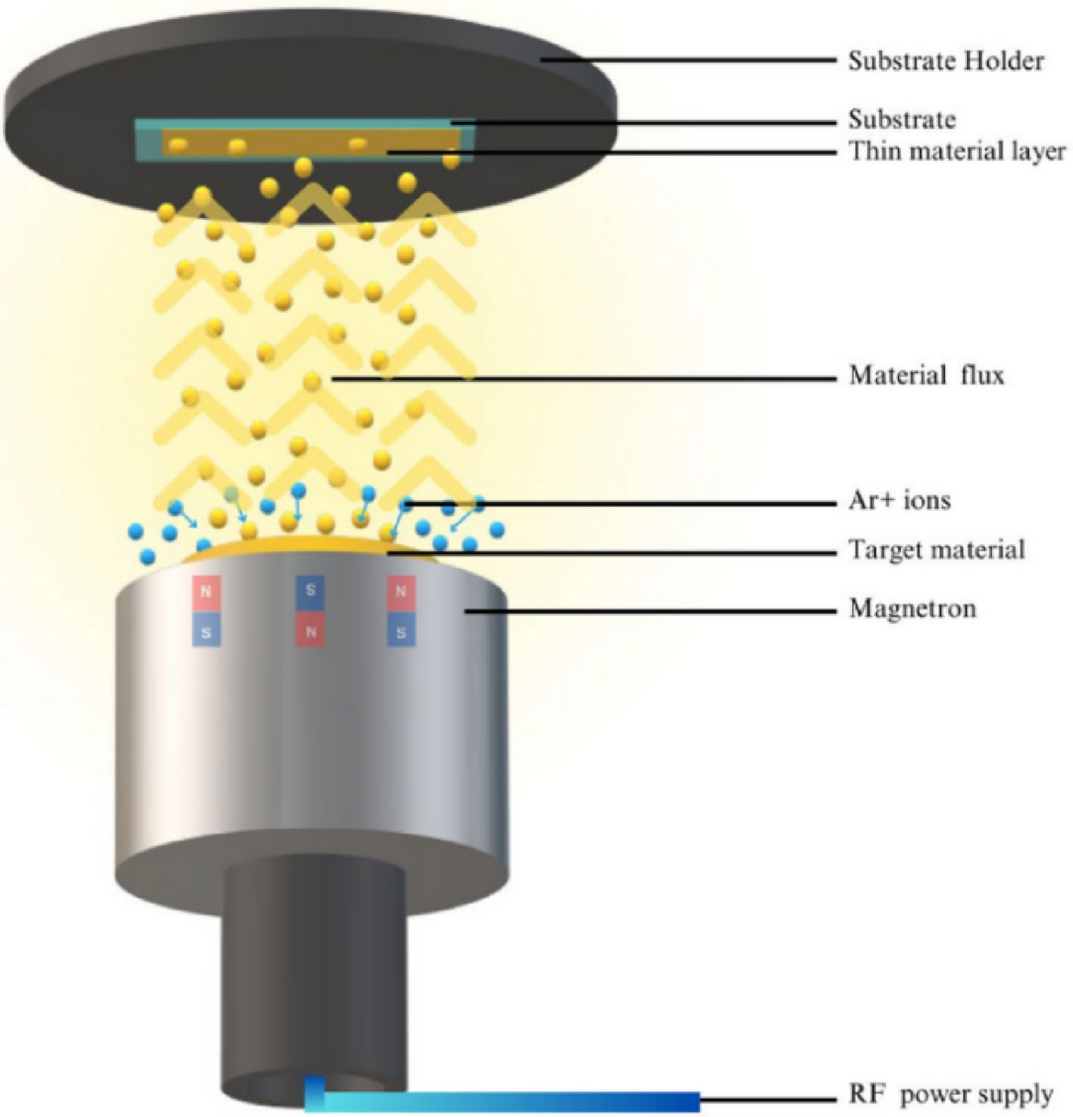
RF magnetron sputtering for thin film deposition [60]

Radio frequency (RF) sputtering is a thin film deposition process that employs radio frequency energy for sputtering material from a target upon a substrate, resulting in nanocomposite films. To prevent interference from air molecules, the whole process is carried out in a vacuum environment as presented in Fig. 9. The substrate is selected carefully based on the purpose and can be made of a range of materials, including glass and silicon. RF sputtering provides benefits such as accurate control of layer thickness, composition, and homogeneity. Ag-Cu_2_O-CuO films were formed onto different substrates, Si(100) and silica glass, at room temperature, using RF-sputtering with a power of 50 W in Ar gas [61].

*G. Regmi et al.* [62] utilized the RF sputtering technique to deposit functional GO/TFN at a power of 110 W and a pressure of 12 m Torr in Ar gas [62]. Co-CuO TFNs were fabricated on various substrates through the RF sputtering process, employing a pure metallic Cu partially coated with a Co target at 150 W in Ar/O_2_ gases. The resulting Co-CuO nanocomposite films exhibit potential suitability as selective absorber layers in solar cell technology [63].

A variety of deposition methods are commonly employed in the production of thin film nanocomposites (TFNs). Each method has its own unique benefits and drawbacks. As demonstrated in Table 1, a comparative analysis is presented of the most frequently utilized techniques, emphasising their typical film characteristics, scalability, cost, and appropriateness for different TFN applications. This analysis facilitates the evaluation of the relative merits of each method, thereby enabling the selection of the most appropriate technique for the intended application in solar energy conversion or water treatment.

**Table 1:** Comparison of common deposition methods for thin film nanocomposites (TFNs) [7, 64]

Deposition method	Typical film properties	Scalability	Cost	Suitability for TFN applications
Spin coating	Smooth and uniform thin films; thickness controlled by spin speed and solution viscosity	Limited (small substrates)	Low	Lab-scale studies, optoelectronic films, photocatalytic thin layers
Chemical bath deposition (CBD)	uniform coatings; low-temperature processing	High (scalable, simple setup)	Low	Photocatalysts, solar absorbers, water treatment coatings
Hydrothermal method	High crystallinity, controlled morphology, good particle dispersion	Moderate (lab to pilot-scale)	Low–moderate	Photocatalysts, metal oxide films, energy and water applications
Spray pyrolysis	Porous, adherent, large-area films; composition tunable by precursor chemistry	High (large-area, simple setup)	Low	Solar cells (photoelectrodes), photocatalysts, antimicrobial coatings
Chemical vapor deposition (CVD)	High-quality, dense, conformal films with good adhesion and uniformity	High (industrial scale)	High	photovoltaic absorbers, barrier coatings
RF sputtering	Dense, uniform, adherent films; good compositional control; suitable for complex oxides	High (industrial and lab scale)	High	photovoltaics, multifunctional coatings

## Thin film nanocomposites application

Recently, both industry and academics have developed a strong interest in nanocomposite films. Nanocomposite films find applications across various fields due to their unique properties and versatility. Nanocomposite films have been employed in drug delivery systems, tissue engineering, and medical imaging, utilizing their biocompatibility and controlled release features to make substantial contributions to healthcare and biomarkers [65, 66]. Nanocomposite film coatings function in industries such as automotive, aerospace, and marine by providing enhanced hardness, wear resistance, and corrosion protection for varied surfaces. Nanocomposite films are valuable in electronic devices such as thin-film transistors, flexible screens, and electronic packaging because of their tailored electrical characteristics and mechanical flexibility. Nanocomposite films are important in solar cell technology because they improve light absorption, charge transfer, and stability, hence increasing solar cell efficiency [7]. In the next part, some of the essential qualities desirable in TFNs for solar cell applications, including high conductivity, longer lifetime, flexibility, cost-effectiveness, and enhanced efficiency of the fabricated solar cells will be discussed.

### Solar cell application

Considerable effort has been dedicated to improving the stability and efficiency of solar cells, with an emphasis on material advances, structural modifications, and innovative deposition techniques [67]. The integration of TFN into solar cell technology has the potential for producing more effective, stable, and sustainable energy conversion, particularly when considering the following aspects [25]:

- **Material selection**: Choose nanomaterials with superior properties, such as high carrier mobility, excellent light absorption, and stability. This may include using advanced nanocomposite materials like perovskites, quantum dots, or specific organic-inorganic hybrids [25].
- **Optimization of nanocomposite structure**: Tailor the nanocomposite structure to maximize light absorption and electron-hole separation. This can involve optimizing the arrangement of nanoparticles, controlling their size, and ensuring a uniform distribution within the thin film. Surface Passivation: Implement surface passivation techniques to reduce defects and enhance stability.
- **Interface engineering**: Optimizing the interfaces within nanocomposite structures is critical for enhancing charge transport and mitigating recombination, thereby improving both stability and device efficiency. The enhancement of charge transport is commonly achieved through the establishment of heterojunctions between nanomaterials and the host matrix. The purpose of these designed interfaces is twofold: firstly, to promote the effective separation of photogenerated electron–hole pairs, and secondly, to reduce recombination losses, thereby enhancing carrier collection efficiency.
- **Electrode materials**: Choose appropriate electrode materials that facilitate efficient charge extraction and transport, contributing to overall device efficiency [68].
- **Device architecture optimization**: Explore novel device architectures, such as tandem or multijunction structures, to boost efficiency by capturing a broader spectrum of sunlight [69].

In this regard, porous BaTiO_3_-RGO nanocomposites are produced using a hydrothermal approach with variable RGO concentrations. Under simulated sunlight, the photovoltaic performance of DSSCs with varying concentrations of RGO-loaded BaTiO_3_-RGO nanocomposite was tested. The DSSC constructed using the BaTiO_3_-RGO with 1.5% of RGO achieved the greatest JSC of 10.59 mA/cm^2^, resulting PCE of 4.93%. This PCE was twice as high as that of the DSSC with a single BaTiO_3_ photoanode. The addition of RGO to the BaTiO_3_ promoted effective electron transport from BaTiO_3_ to the FTO substrate, reducing recombination rates of photo-induced charge carrier. The obtained results highlight BaTiO_3_-RGO nanocomposites as interesting alternatives for fabricating effective photoanodes in DSSCs [70]. CeO_2_–CuO nanocomposite films were prepared using chemical processes. The prepared TFNs were utilized as an anode material for the fabrication of PSCs, leading to an achieved PCE of 10.58%.

The enhanced device performance observed for the improved performance of the prepared nanocomposite, in contrast to pure CeO_2_, can be attributed to the distinctive properties of CeO_2_–CuO. These include high hole mobility, favorable energy level alignment with CH_3_NH_3_PbI_3_, and an extended lifetime of photo-excited carriers. These features contribute to improve in the development of industrial-scale PSCs [71]. *Satish Bykkam et al.* [72] studied the impact of incorporating FLG into different metal oxides on the efficiency of DSSCs. The doctor blade method was used to prepare thin films of the metal oxides and FLG/metal oxide nanocomposites. In comparison to other FLG/metal oxide nanocomposites, a notable enhancement of 6.60% in PCE was achieved in DSSCs using FLG/TiO_2_ nanocomposite as the photoanode. The obtained results indicate that FLG in TiO_2_ acts as a blocking layer in DSSCs, inhibiting back recombination of electrons and holes, and thereby enhancing PCE. The FLG/TiO_2_ films emerged as a superior photoanode for DSSCs applications [72]. *K. Pugazhendhi et al.* [73] reported that TiO_2_/Al@ZnO nanocomposites exhibit superior physical properties compared to tradational TiO_2_ and ZnO when serving as photoanodes. The addition of Al^3+^ into ZnO within the TiO_2_/ZnO nanocomposite films resulted in an approximately 78% enhancement in efficiency, attributed to synergistic effects such as plasmonic effects, bandgap change, and nanocomposite formation.

This results in unique properties, including J_SC_ of 33 mA cm^− 2^, V_OC_ of around 0.41 V, FF of approximately 0.81, and a promising efficiency of 11.4%. These findings offer the potential for improving and developing a new class of next-generation plasmonic DSSCs. Additionally, the TiO_2_/Al@ZnO nanocomposite photoanode has the potential for use in PSCs and solar cells [73]. The introducing of GO to ZnO as an EEL considerably increased the performance of PSCs. Incorporating GO improves the penetration of the perovskite precursor into ZnO, resulting in a more uniform perovskite coating that has improved surface area and higher electronic properties. The introduction of GO, along with Ag doping, resulted in the highest PCE of 8.72%, above PSCs based on nanostructured ZnO, which had a PCE of roughly 7.27%. This enhancement is attributable to increased charge collection at the interface, lower trap-assisted recombination probability, and improved light absorption and charge transfer. The synergistic impact of GO integration with Ag doping in the ZnO structure confirms them as effective components for EEL in PSCs, particularly at lower temperatures [74].

*G. Regmi et al.* [75] established a novel trimetallic oxide nanocomposite coating called ZZTO, which is composed of ZrO_2_, ZnO, and TiO_2_ and was deposited using RF-sputtering. This nanocomposite was used as an anti-reflective coating on CuInSe_2_ thin film solar cells, with additional benefits such as self-cleaning and anti-corrosion. The ZZTO coating has outstanding optical characteristics, high transmittance, and remarkable hydrophobic and corrosion-resistant properties. Solar cells constructed on flexible substrates, with and without anti-reflective coating, were evaluated to validate multifunctionality and photoconversion efficiency enhancements. The reduction in optical loss resulted in increased light absorption across a wide spectrum, raising J_SC_ from 31.04 to 33.05 mA/cm^2^ and enhancing effectiveness from 9.11% to 10.24%. The developed ZZTO anti-reflective coating exhibited superhydrophobicity, high transparency, and corrosion-resistant features. Thus, the multifunctional ZZTO coating has the potential to revolutionize anti-reflective materials and become an ideal choice for solar cell technology [75]. PANI-Fe_3_O_4_-TiO_2_ nanocomposite was synthesized via a basic solid-state chemical process. An FTO/TiO_2_/PANI-Fe_3_O_4_-TiO_2_/Al cell was fabricated to test photovoltaic characteristics The resultant device displayed good photovoltaic efficiency, with a PCE of 1.74%. This is an improvement over solar cells based on nanocomposites’ absence of Fe_3_O_4_ (PANI-TiO_2_) or TiO_2_. The existence of Fe_3_O_4_ is responsible for increased cell performance, as it serve to the blocking of the recombination process [76]. *M. Chakraborty et al.* [77] developed nanostructured ZnO covered with TiO_2_-GO nanocomposites using a low-cost chemical process, demonstrating their use as photoanodes in DSSCs. TiO_2_ thin films containing different concentrations of GO were spin-coated onto vertical ZnO nanowire arrays. The hybrid nanostructure-based DSSC demonstrated higher η than a pure ZnO photoanode. As the amount of GO in the TiO_2_-GO nanocomposite layers deposited on the nanostructured ZnO raised correspondingly increased the improvement in essential measurements. The hybrid nanostructure of ZnO NW/TiO_2_-GO (10%) showed excellent performance with η ~ 0.72%, V_oc_ ~491 mV, J_sc_ ~2.19 mA/cm^2^, and FF ~ 0.66. This enhancement in performance is attributed to the combined effects of improved charge transport along the ZnO nanowire, increased contact area, and conductivity modulation of TiO_2_ through the inclusion of a conductive GO network. The results indicate that a specific amount of GO significantly alters the interfacial effects in the fabricated hybrid nanostructures, facilitating improved charge transportation [77]. *I.H.K*. *Madigasekara et al.* [78] successfully synthesized an Ag-AgBr-TiO_2_ nanocomposite and used it in DSCs, providing an 8.46% improvement in PCE. This demonstrates a significant 31% increase over the reference cell constructed using TiO_2_, which had a PCE of 6.45%. The increased efficiency can be attributable to the combined actions of AgBr and metallic Ag. Under approximately 470 nm visible light, AgBr is highly efficient in generating electrons and holes. The enhanced effects provided by plasmonic effect of Ag nanoparticles promote charge production and separation in both the dye and AgBr. These combined effects, together with the stability given by the TiO_2_ structure of the DSC active material, result in an effectively functioning DSCs due to the integration of the Ag/AgBr/TiO_2_ nanocomposite in solar cell field [78]. *Anindita Chatterjee et al.*. used co-precipitation to create ZnO/CuO nanocomposites with variable ZnO and CuO ratios. The ZnO/CuO nanocomposite with a 1:1 ratio showed a conversion efficiency of 7.62% when compared to ZnO and CuO alone. The increased efficiency can be related to the creation of a p-n heterojunction by CuO/ZnO nanocomposites, which increases the number of electrons and holes participating in surface conduction.in addition, the ZnO/CuO nanocomposite absorbs a wide range of solar spectra, increasing light conversion efficiency [79]. Ag-NiO_x_ nanocomposites thin films were prepared and inserted into the absorber layer of bulk-heterojunction solar cells to compare with undoped devices The ideal nanocomposite concentration for the greatest device performance with a PCE of 5.13% and a FF of 47%. The obtained results represent a remarkable improvement in PCE against the reference solar cell. The prepared Ag-NiO_x_ nanocomposites are highly stable under ambient circumstances and may be manufactured using roll-to-roll technology, potentially lowering the cost of solar panels [80].

Perovskite solar cells have demonstrated potential for high PCE, however, obstacles such as short lifetime and poor stability prevent their commercialization. To address this issue, interface engineering of the ETL with 2D nanostructured materials has attracted interest as a result of their thermal stability, and variable work function, which have a substantial influence on charge carrier dynamics. *Se-Phin Cho et al.* [81] demonstrated a cost-effective method for producing a solution-processable nanocomposite of rGO and PVP. This nanocomposite worked well as a HTL in PSCs, ensuring dispersion stability for six months. Importantly, PSCs using the RGO/PVP nanocomposite demonstrated higher PCE and device stability than those using the standard PEDOT: PSS comparable. This emphasizes the RGO/PVP nanocomposite’s promise as a cost-effective and scalable option for durable, high-performance PSCs [81]. *Ramesh Banoth et al.* [82] developed an efficient strategy to improve the PCE and air stability of PSCs by adding 2D-MoS_2_ into mp-TiO_2_ nanocomposites. The best solar cell, with c-TiO_2_/mp-TiO_2_:MoS_2_ (0.5 wt%), demonstrated a significant improvement in PCE to ~ 13.04%, compared to the reference device with PCE ~ 8.75%, proving the benefit of incorporating MoS_2_ in the mp-TiO_2_ as buffer layer. Introducing nanostructured MoS_2_ to mp-TiO_2_ increased PCE by ~ 49% and increased lifetime, keeping ~ 86% of the starting value for 500 h. The reported results introduce mp-TiO_2_:2D-MoS_2_ as an efficient ETL for high-performance, low-cost, air-processed PSCs, with promising Prospective progress in solar cell applications [82].

To enhance the efficiency of DSSCs, charge carrier production and transportation must be enhanced to prevent charge recombination. *M. Mohammadnezhad et al.* [83]. constructed a fast and easy nanocomposite using AuNPs and MWCNTs. This nanocomposite was applied to mp-TiO_2_ films used as photoanodes in DSSCs to improve light absorption and charge carrier production. The AuNP/MWCNT incorporated directly into the TiO_2_ working electrode, achieved PCE of 6.61% and a J_sc_ of 12.26 mA cm^− 2^. This resulted in a ~ 31% increase in PCE compared to DSSCs using TiO_2_ alone. Furthermore, DSSCs with the TiO_2_/AuNP/MWCNT photoanode demonstrated significant stability, keeping 92% of the first PCE value even after continuous lighting for 10 days, compared to the reference cell [83]. Gr-based nanocomposites in the photoanode contribute significantly to solar cell efficiency by assisting in photon absorption and charge separation. In this regard, *Savisha Mahalingam et al.* reported that the use of ZnO coupled with Gr as the photoelectrode in DSSCs, resulted in enhanced performance. The resulting ZnO/Gr-based solar cells achieved an efficiency of up to 11.5% using different dyes and electrolytes [84]. In view of the recent advancements outlined above, Table 2 offers a synopsis of the critical performance metrics of thin film nanocomposite (TFN) systems employed in solar cell applications, systematised according to their synthesis methodologies.

**Table 2:** Performance metrics of thin film nanocomposite (TFN) systems for solar cell applications by synthesis technique

TFN system	Synthesis method	Application type	PCE (%)	Ref.
BaTiO₃–RGO nanocomposites	Hydrothermal + spin	DSSC	4.93	[70]
CeO₂–CuO nanocomposites	Chemical deposition	PSC	10.58	[71]
FLG–TiO₂ nanocomposites	Doctor-blade	DSSC	6.60	[72]
TiO₂/Al@ZnO nanocomposites	Chemical process	DSSC/PSC	11.40	[73]
GO–Ag doped ZnO	Spin-coating	PSC	8.72	[74]
ZZTO (ZrO₂–ZnO–TiO₂) nanocomposites	RF sputtering	CIS solar cells	10.24	[75]
Ag/AgBr–TiO₂ nanocomposites	Chemical process	DSC	8.46	[78]
ZnO–CuO nanocomposites (1:1)	Co-precipitation	Solar cells	7.62	[79]
MoS₂–TiO₂ nanocomposites	Solution process	PSC	13.04	[82]
ZnO–Graphene nanocomposites	Solution process	DSSC	11.50	[84]

### Water purification

TFNs exhibit remarkable effectiveness in water purification due to their high reactivity and photocatalytic properties [85]. Ensuring access to clean water is vital for public health, and the use of nanocomposites offers innovative approaches to tackle water contamination challenges [86, 87].

**Potential benefits in water treatment**

TFNs represent a versatile strategy for water treatment, providing effective solutions for the degradation of pollutants, removal of heavy metals, and control of pathogens [85, 88]. Their elevated reactivity and selectivity allow for the efficient and sustainable management of intricate water contamination challenges. The unique characteristics of TFNs, including a high surface area, improved catalytic activity, selective adsorption capabilities, and antimicrobial properties, have attracted significant interest in the field of water treatment (Table 3). These films are produced by integrating nanoparticles such as metal oxides, metal sulfides, carbon-based nanomaterials, or polymers into a resilient thin matrix, which can be customized to target specific water contaminants [85, 88]. A detailed examination of the applications of TFNs in water treatment (Table 3) follows.

- **Photocatalytic degradation of pollutants**: TFNs incorporating metal oxide nanoparticles, including titanium dioxide (TiO₂) and zinc oxide (ZnO), exhibit remarkable photocatalytic efficiency. Upon exposure to ultraviolet or visible light, these materials produce reactive oxygen species (ROS) capable of degrading organic contaminants, such as dyes, pharmaceuticals, and pesticides, into harmless byproducts. Consequently, TFNs are particularly effective in facilitating advanced oxidation processes (AOPs) for the treatment of water [89–91].
- **Antibacterial properties**: TFNs that incorporate silver, copper, or zinc oxide nanoparticles demonstrate significant antibacterial properties, making them particularly effective in inhibiting biofilm development and eradicating pathogens in aquatic environments. The antimicrobial characteristics of these materials are advantageous for their use in the purification of drinking water and the treatment of wastewater, where the management of pathogens is crucial [85, 88].
- **Membrane fouling resistance**: In the context of water treatment, membrane fouling, which results from the buildup of organic substances, bacteria, and mineral deposits, presents a significant challenge [92]. The application of TFNs as coatings on filtration membranes has been shown to mitigate fouling by integrating hydrophilic nanoparticles that effectively repel contaminants. This enhancement not only extends the operational lifespan of the membranes but also decreases maintenance requirements, thereby contributing to a reduction in overall operational expenses [93–95].

**Table 3:** Applications of thin-film nanocomposite (TFN) membranes in water treatment, categorized by target contaminant class, incorporated nanomaterials, applied technology

Application area	Nanomaterials used	Performance summary	Specific contaminants treated	Application	Ref.
Desalination	CNTs, GO, MOFs	Enhanced water flux, better salt rejection, reduced fouling	Salts, seawater, brackish water	Reverse Osmosis (RO), Forward Osmosis (FO)	[101, 102]
Industrial wastewater	TiO_2_, NiS_2_, Sb_2_S_3_	organic pollutants photodegradation	Methylene Blue, Rhodamine B, phenol, acetaminophen and paraquat	Photocatalysis	[89–91]
Municipal wastewater	Ag nanoparticles, CNTs, GO	Removal of micropollutants, pathogen inactivation	Pharmaceuticals, personal care products, pathogens	Membrane Bioreactor (MBR), Ultrafiltration (UF)	[103, 104]
Drinking water production	GO, Silica nanoparticles	Safe drinking water, antimicrobial properties	Bacteria, viruses, chemical pollutants	Microfiltration (MF), MF Nanofiltration (NF)	[103, 105]
Emerging contaminants	CNTs, GO	Effective removal of PPCPs, EDCs	Pharmaceuticals, personal care products, endocrine-disrupting chemicals	Advanced Oxidation Processes (AOPs)	[106]
Pathogen disinfection	Ag nanoparticles, ZnO,	Inactivation of pathogens, reduced biofouling	Bacteria, viruses, protozoa	Photocatalysis, Electrochemical Disinfection	[96, 97]

### Disinfection using reactive oxygen species (ROS)

Reactive Oxygen Species (ROS) are highly reactive oxygen-containing molecules, including peroxides, superoxide, hydroxyl radicals, and singlet oxygen. Their potent oxidizing capabilities render them effective agents for disinfection. When integrated with TFNs, ROS can be utilized to develop efficient and sustainable disinfection methods [96, 97].

Mechanism of ROS-Induced Disinfection.

- **Oxidative stress**: The presence of ROS can induce oxidative stress within microbial cells, resulting in damage to cell membranes, proteins, and DNA, which may ultimately lead to cell death.
- **Membrane disruption**: ROS can compromise the structural integrity of microbial cell membranes, resulting in the leakage of cellular contents and subsequent cell lysis.
- **DNA damage**: ROS can cause breaks in DNA strands, hindering microbial replication and contributing to cell mortality.
- **Protein denaturation**: ROS can oxidize and denature critical proteins, disrupting essential metabolic functions in microbes.

### Desalination and Ion-exchange applications

Ion-Selective TFNs can be designed to enable the preferential transport of particular ions, thereby improving desalination processes and the extraction of specific ions [98]. This mechanism is based on integrating nanoparticles, such as zeolites, metal-organic frameworks (MOFs), or ion-exchange resins, into thin films, which facilitate selective ion exchange or adsorption, making them beneficial for desalination purposes. A prominent instance of this technology is the emergence of MOF-based TFNs, which are increasingly recognized for their capacity to recover lithium from brines and seawater selectively. This development presents a promising opportunity for desalination technologies to not only reclaim valuable ions but also to remove unwanted ones effectively [99].

### Advantages of Thin film nanocomposite in water treatment

- **Enhanced durability**: These films present a robust, enduring surface suitable for ongoing water treatment applications [100].
- **High surface area**: The incorporation of nanoparticles results in a significantly larger active surface, facilitating enhanced interactions with various contaminants [100].
- **Customizability**: A variety of nanoparticles and polymers can be integrated to target specific types of pollutants [100].
- **Energy efficiency**: Numerous processes utilizing nanocomposites, including photocatalysis, can harness solar energy, thereby promoting energy efficiency [100].

## Future directions and challenges for thin film nanocomposites

TFNs represent an innovative technology with potential applications across various domains, such as solar energy conversion and water purification. The distinctive characteristics of these materials arise from the synergistic interactions among their constituent elements, resulting in improved performance and enhanced functionality.

**Future directions**:

- **Enhanced efficiency and stability in solar cells**:
  - **Multi-junction solar cells**: TFNCs can be employed in the development of multi-junction solar cells, which are capable of capturing a wider range of the solar spectrum, consequently enhancing their efficiency.
  - **Perovskite solar cells**: The incorporation of nanomaterials into perovskite solar cells has the potential to enhance both their stability and efficiency.

- **Advanced water treatment technologies**:
  - **Photocatalysis**: TFNCs can serve as effective photocatalysts for the degradation of organic pollutants and the disinfection of water when exposed to solar light.
  - **Membrane filtration**: Nanocomposite membranes have the potential to improve both the efficiency and selectivity of filtration processes in water purification applications.

**Challenges and environmental and economic impact:**

- **Scalability and cost-effectiveness**:
  - Increasing the production capacity of TFNCs while ensuring quality and uniformity presents a considerable challenge.
  - The establishment of economic synthesis techniques is essential for their broader acceptance.

- **Long-term stability and durability**:
  - Ensuring the sustained stability and longevity of TFNC-based devices across diverse environmental conditions is of paramount importance.
  - It is vital to comprehend the mechanisms of degradation and to formulate strategies aimed at their mitigation.

- **Environmental impact**:
  - It is essential to thoroughly evaluate the environmental consequences associated with the synthesis and disposal of TFNCs.
  - Furthermore, the advancement of sustainable and environmentally friendly synthesis methods is of significant importance.

- **Economic impact**:
  - The extensive implementation of TFNC-based technologies has the potential to generate considerable economic benefits, leading to the establishment of new industries and the creation of employment opportunities.
  - Nevertheless, the challenges associated with initial capital expenditures and intellectual property rights may hinder this progress.

## Conclusion and future prospects

The incorporation of nanomaterials into thin film nanocomposites (TFNs) has emerged as a promising approach to enhance the efficiency of solar cells and water treatment technologies. The present essay will offer a comprehensive review of the relevant literature on this topic. The composites under scrutiny have demonstrated superior characteristics, including enhanced light absorption, elevated charge carrier mobility, and augmented catalytic activity, in comparison to their constituent materials. Notwithstanding these advances, there are several significant challenges that still need to be addressed, including issues related to large-scale production, cost-effectiveness, long-term durability, and potential environmental consequences. In the domain of solar energy, the power conversion efficiency (PCE) of TFN-based solar cells is contingent on the fabrication techniques employed and the selection of constituent materials. Recent research has indicated that organic-based nanocomposites, which integrate polymers with nanoparticles, demonstrate improved flexibility and tunability. In contrast, inorganic-based systems, including metal oxides and quantum dot composites, have demonstrated enhanced stability and higher efficiency. The present essay will offer a comprehensive review of the relevant literature on this topic. In the domain of water treatment applications, TFNs have been demonstrated to enhance the photocatalytic degradation of pollutants and improve antimicrobial performance. This offers a pragmatic solution for sustainable clean water technologies. The versatility of TFNs also allows for the customization of properties to fulfil specific needs, including charge recombination control in solar cells and surface reactivity in water treatment membranes.

The future progress of this domain is contingent on the development of environmentally sustainable and scalable deposition techniques, in conjunction with comprehensive assessments of material stability and performance in realistic operating environments. It is anticipated that advancements in nanostructure engineering and the production of hybrid materials will enhance both the optical and catalytic properties of these materials. Notwithstanding the challenges that persist, a persuasive rationale exists to substantiate the proposition that TFNs have the potential to make a substantial contribution to the development of next-generation solar energy systems and water purification technologies.

## Data Availability

All data generated or analyzed during this study are included in this published article.
